# MULTIPLE METHODS OF ASSESSING DAILY MEDIA USE IN LATE LIFE

**DOI:** 10.1093/geroni/igz038.1532

**Published:** 2019-11-08

**Authors:** Karen Fingerman, Crystal L Ng, Meng L Huo, Shiyang L Zhang

**Affiliations:** 1 University of Texas, Austin, Austin, Texas, United States; 2 The University of Texas at Austin, Austin, Texas, United States

## Abstract

Television viewing is a risk factor for obesity and poor physical health. By contrast, close ties to family and friends in late life are often beneficial. This study examined associations between social engagement and television viewing. Participants (N = 313) from the Daily Experiences and Well-being Study completed an initial interview about their social partners and participated in a 5 to 6 day intensive data collection including Ecological Momentary Assessments about their social contact and activities every 3 hours. Participants also wore Electronically Activated Recorders (EAR) which captured snippets of sound in the environment. Multilevel models using self report and EAR data revealed that participants were more likely to watch TV when they were with close family members (e.g., spouse, grown children) than with friends or acquaintances. Findings from these multiple methods suggest that close family may encourage risks (e.g., sedentary behaviors) as well as benefits in late life.

